# SE‐lncRNAs in Cancer: Classification, Subcellular Localisation, Function and Corresponding TFs

**DOI:** 10.1111/jcmm.70296

**Published:** 2024-12-17

**Authors:** Yuxin Bao, Songling Teng, Hanjie Zhai, Yuanzhuang Zhang, Yeqiu Xu, Chenghao Li, Zhenjun Chen, Fu Ren, Yong Wang

**Affiliations:** ^1^ Fourth Department of Orthopaedic Surgery Central Hospital Affiliated To Shenyang Medical College Shenyang Liaoning P. R. China; ^2^ Department of Hand Surgery Central Hospital Affiliated To Shenyang Medical College Shenyang Liaoning P. R. China; ^3^ Department of Neurosurgery Central Hospital Affiliated To Shenyang Medical College Shenyang Liaoning P. R. China; ^4^ Department of Anatomy, School of Basic Medicine Shenyang Medical College Shenyang Liaoning P. R. China

**Keywords:** cancer, ECM remodelling, SE‐lncRNAs, subcellular location, transcriptional regulation

## Abstract

Emerging evidence highlights certain long noncoding RNAs (lncRNAs) transcribed from or interacting with super‐enhancer (SE) regulatory elements. These lncRNAs, known as SE‐lncRNAs, are strongly linked to cancer and regulate cancer progression through multiple interactions with downstream targets. The expression of SE‐lncRNAs is controlled by various transcription factors (TFs), and dysregulation of these TFs can contribute to cancer development. In this review, we discuss the characteristics, classification and subcellular distribution of SE‐lncRNAs and summarise the role of key TFs in the transcription and regulation of SE‐lncRNAs. Moreover, we examine the distinct functions and potential mechanisms of SE‐lncRNAs in cancer progression.

## 1 Introduction

Cancer is a localised result of abnormal cellular behaviour at the genetic level, marked by a loss of normal growth regulation. It arises from changes in cell proliferation and gene expression that disrupt cellular homeostasis and lead to uncontrolled growth. Essentially, cancer is a genetic disease characterised by defects in cell differentiation and growth [1]. Recent advances in genome sequencing and functional genomics have led to the discovery of numerous genomic elements whose mutations, changes in expression or epigenetic modifications serve as ‘cancer markers’ [2]. Research progress has improved our understanding of the molecular causes of cancers, leading to significant advances in its diagnosis and treatment. Despite these advances, the number of cancer cases continues to increase each year, highlighting the need to explore additional internal mechanisms driving cancer development [3].

The advent of next‐generation sequencing technologies has led to the discovery of numerous noncoding RNAs (ncRNAs), which account for nearly 60% of the transcriptional output in human cells [4]. Long noncoding RNAs (lncRNAs), a major subclass of the ncRNA family, are RNA transcripts longer than 500 nt that do not encode proteins [5, 6]. However, this is merely a broad definition, as it includes all transcripts that lack obvious protein‐coding potential. Interestingly, some lncRNAs previously classified in this way have been found to encode micropeptides, revealing unexpected layers of complexity [5, 7]. LncRNAs regulate gene expression and function at multiple levels. LncRNAs can regulate the expression of nearby genes in *cis* and distant genes in *trans*, participate in RNA metabolism, participate in translation and modulate chromatin structure [8]. Via a multitude of anchor DNA sites, lncRNAs can interact with DNA by forming an RNA:DNA:DNA triplex, thereby contributing to the local chromatin organisation of super‐enhancers (SEs) [9].

SEs, first proposed by Richard A. Young in 2013, are much larger than general enhancers: SEs are clusters of enhancers that span large regions of DNA with a high concentration of transcriptional coactivator binding [10]. SEs exhibit a number of distinctive features that account for their unique biological properties. SEs are dependent on topologically associating domains (TADs) with higher order structure [11, 12]. The CCCTC‐binding factor (CTCF) is a highly conserved zinc‐finger protein that defines TAD boundaries and isolates SEs from adjacent regions. Recent studies have shown that SEs can function as membrane‐free, phase‐separated compartments in the nucleus, where they encapsulate and thereby stabilise robust transcription [12]. Additionally, the mediator complex, RNAPII, bromodomain‐containing protein 4 (BRD4) and active chromatin markers (e.g., H3K27ac and H3K4me1) were significantly enriched in the SE region [13].

Recently, the concept of super‐enhancer–associated long noncoding RNAs (SE‐lncRNAs) has been frequently discussed, although the literature is often unclear on the topic. Enhancer RNAs (eRNAs), transcribed from active enhancers, are characterised by their bidirectional capping, relatively short length, lack of splicing, lack of polyadenylation and rapid degradation [8, 14, 15]. In contrast to eRNAs, SE‐lncRNAs specifically refer to lncRNAs that are transcribed from or interact with SEs. The transcriptional activity of SE‐lncRNAs heavily depends on SEs [9, 16]. SE‐lncRNAs are commonly unidirectional, polyadenylated and spliced [8].

SE‐lncRNAs are associated with a wide range of human diseases, and research has focused particularly on their role in tumorigenesis and cancer progression [17, 18, 19]. SE‐lncRNAs regulate gene expression by interacting with long‐range chromatin or chromatin loops or by modulating SE activity to influence both normal and abnormal physiological processes [20, 21]. Currently, dynamic epigenetic alterations such as super‐enhancer (SE) hijacking events are recognised as key factors leading to metastasis [22]. The functional roles of SE‐lncRNAs in cancer are complex and multifaceted. While substantial evidence supports the oncogenic functions of SE‐lncRNAs across various cancers, there are exceptions. For example, the SE‐lncRNA RP11‐569A11.1 is downregulated in colorectal cancer (CRC), and its overexpression has been shown to suppress CRC progression by increasing the expression of interferon‐induced protein with tetratricopeptide repeats 2 (IFIT2) [23].

In contrast to previous reviews that examined only the role of SE‐lncRNAs in cancer, in this review, we comprehensively summarise the characterisation, subcellular distribution and functional mechanisms of SE‐lncRNAs by exploring the roles of SE‐lncRNAs in different cancer types.

## 2 Cis‐Acting and Trans‐Acting SE‐lncRNAs

**FIGURE 1:**
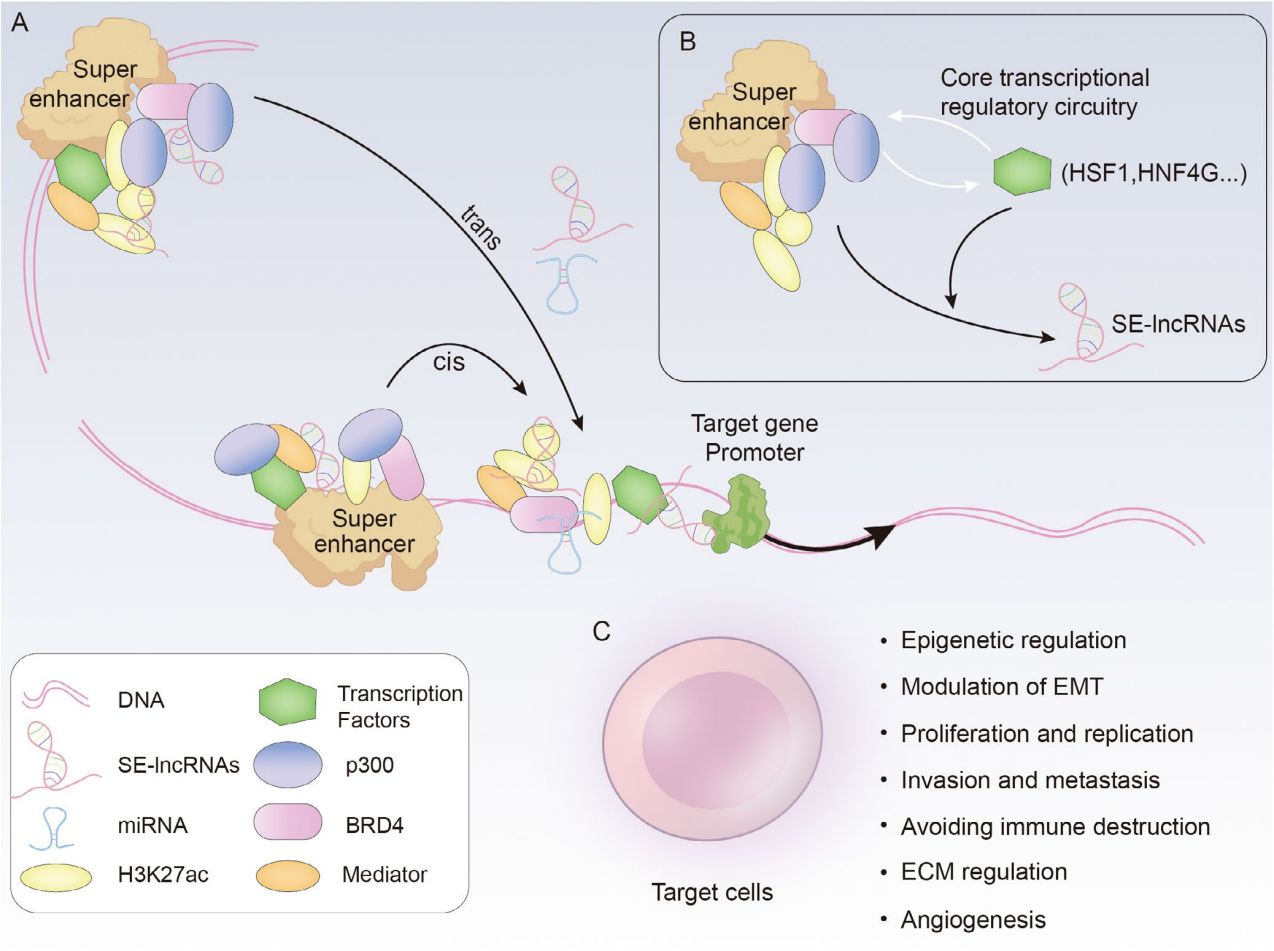
(A) Schematic representation of *cis*‐acting and *trans*‐acting SE‐lncRNAs with distinct mechanisms of action. (B) The associated TFs establish a core transcriptional regulatory circuitry for transcriptional activity in conjunction with SEs. (C) SE‐lncRNAs exert multiple functions on target cells.

LncRNAs are known to operate across multiple levels, including epigenetic modification, transcription, translation and posttranslational processes [6]. SE‐lncRNAs, a specific class of lncRNAs mapped to SEs, can be categorised on the basis of their functional mechanisms. *Cis*‐acting SE‐lncRNAs are transcribed from SEs and regulate nearby genes, whereas *trans*‐acting SE‐lncRNAs are transcribed from other genomic locations and interact with SEs to regulate distant genes (Figure 1A) [20, 24].

### 2.1 *Cis*‐Acting SE‐lncRNAs

*Cis*‐acting SE‐lncRNAs influence the activity of neighbouring genes by interacting with enhancer sequences, achieving *cis*‐regulation through intrachromosomal interactions [25]. As a *cis*‐acting SE‐lncRNA, pluripotency‐associated transcript 22 (Platr22) functions as a pivotal regulator of mouse embryonic stem cell (mESC) pluripotency maintenance. Platr22 regulates the transcriptional activity of its associated SE, ^Platr22^SE. By interacting with DEAD‐box helicase 5 (DDX5) and heterogeneous nuclear ribonucleoprotein L (hnRNPL), Platr22 modulates pluripotency in mESCs by regulating the expression of its nearby gene, zinc finger protein 281 (ZFP281), a crucial pluripotency regulator [26].

Additionally, the SE‐lncRNAs RP11‐379F4.4 and RP11‐465B22.8 operate in a *cis*‐acting manner by modulating the expression of nearby genes and have been identified as potential indicators of progression from ductal carcinoma in situ (DCIS) to invasive ductal carcinoma (IDC) [27].

### 2.2 Trans‐Acting SE‐lncRNAs

Extended open‐chromatin regions that are highly transcribed at SEs may produce RNAs with enough abundance and stability to perform broad functions *in trans* [24].

For enhancer RNAs (eRNAs), *cis*‐regulation of target loci through intrachromosomal interactions is considered more common than *trans*‐regulation via interchromosomal interactions [25, 28]. These findings suggest that eRNAs function primarily through intrachromosomal interactions rather than through interchromosomal interactions [25]. However, it is still uncertain whether SE‐lncRNAs use the same regulatory mode.

Pax7‐associated muscle lncRNA (PAM) is an SE‐lncRNA that primarily interacts with many genomic sites in *trans*. It regulates muscle satellite cell (SC) activity by binding to DEAD‐box helicase (DDX) and facilitating chromatin interactions between the PAM on the SE and target genes [25]. Similarly, the SE‐lncRNA Bloodlinc functions in *trans* to regulate gene expression. Bloodlinc, which is transcribed from the maternal SE, spreads to multiple *trans*‐chromosomal loci outside the structural domain of the maternal SE and plays a role in regulating erythropoiesis [24].

## 3 Relationship Between the Subcellular Localisation and Function of SE‐lncRNAs

The subcellular distribution of lncRNAs is crucial for determining their molecular functions [29]. Thus, the functional mechanism of SE‐lncRNAs is intricately linked to their intracellular positioning.

### 3.1 SE‐lncRNAs in the Nucleus

In the nucleus, certain SE‐lncRNAs modulate transcriptional programmes through interactions with chromatin and chromatin remodelling. They also act as scaffolds to help establish the spatial organisation of the nuclear compartment [30]. Nuclear SE‐lncRNAs exhibit significantly enhanced functionality in transcriptional regulation and chromatin interactions [31]. Both *cis*‐acting and *trans*‐acting SE‐lncRNAs are most likely localised to the nucleus [20, 27].

The lncRNA metastasis‐associated lung adenocarcinoma transcript 1 (MALAT1), which is found primarily in the nucleus, is an SE‐lncRNA [30, 32]. MALAT1 promotes c‐myc‐mediated epithelial–mesenchymal transition and promotes epithelial ovarian cancer (EOC) progression by acting as a miR‐22 sponge [33].

### 3.2 SE‐lncRNAs in the Cytoplasm

In contrast, some SE‐lncRNAs are found in the cytoplasm, where they can influence gene expression through different mechanisms. Cytoplasmic SE‐lncRNAs can interact with diverse protein partners, thereby influencing RNA stability, degradation, translation and mRNA splicing [34].

In contrast to nuclear SE‐lncRNAs, cytoplasmic SE‐lncRNAs perform their biological functions through several mechanisms. First, they can interact with TFs to establish a positive feedback loop that promotes carcinogenesis. Second, cytoplasmic SE‐lncRNAs can influence posttranslational modifications by acting as competing endogenous RNAs (ceRNAs) or molecular sponges. This interaction alters the availability of microRNAs (miRNAs), thereby regulating target gene expression. Additionally, cytoplasmic SE‐lncRNAs play a role in cancer progression by mediating signal transduction pathways [31]. For example, the SE‐lncRNA LINC01503, regulated by the tumour protein p63 (TP63), activates the ERK/MAPK and PI3K/Akt signalling pathways, thereby increasing the proliferation and invasion of oesophageal squamous cell carcinoma (ESCC) cells [19].

The subcellular localisation of SE‐lncRNAs in the cytoplasm underscores their versatile roles beyond nuclear functions, highlighting their importance in the posttranscriptional regulation of gene expression.

However, the localisation of SE‐lncRNAs is not fixed. The subcellular localisation of lncRNAs is also associated with alternative polyadenylation signals [8]. For example, the SE‐lncRNA colon cancer‐associated transcript 1 (CCAT1) exists in two isoforms: the long isoform (CCAT1‐L), which is predominantly localised to the nucleus, and the short isoform (CCAT1‐S), which is mainly found in the cytoplasm [35, 36].

In summary, SE‐lncRNAs have significant potential for regulating cancer development because of their selective binding to specific targets within various subcellular environments. Understanding the functions of SE‐lncRNAs in different cellular locations offers valuable insights into their roles in cellular processes and disease mechanisms. This highlights the critical importance of their spatial distribution for their regulatory functions.

## 4 TFs Involved in SE‐lncRNA Transcription

SEs have been described as clusters of elements with enhancer bioinformatic signatures that are enriched with particularly high levels of TF recruitment [37, 38]. Specific TFs can bind SEs to regulate gene expression. Some TFs can regulate their own expression as well as other factors in a feed‐forwards loop, collaborating with their associated SEs to establish core transcriptional regulatory circuitry (CRC). This circuitry plays a crucial role in regulating gene expression (Figure 1B) [39, 40, 41]. Accurate interactions among TFs, cofactors and chromatin regulators at genomic regulatory elements, such as SEs, are essential for effective transcriptional progression [42]. These findings suggest that TFs are likely to play important roles in the transcription of SE‐lncRNAs.

**FIGURE 2:**
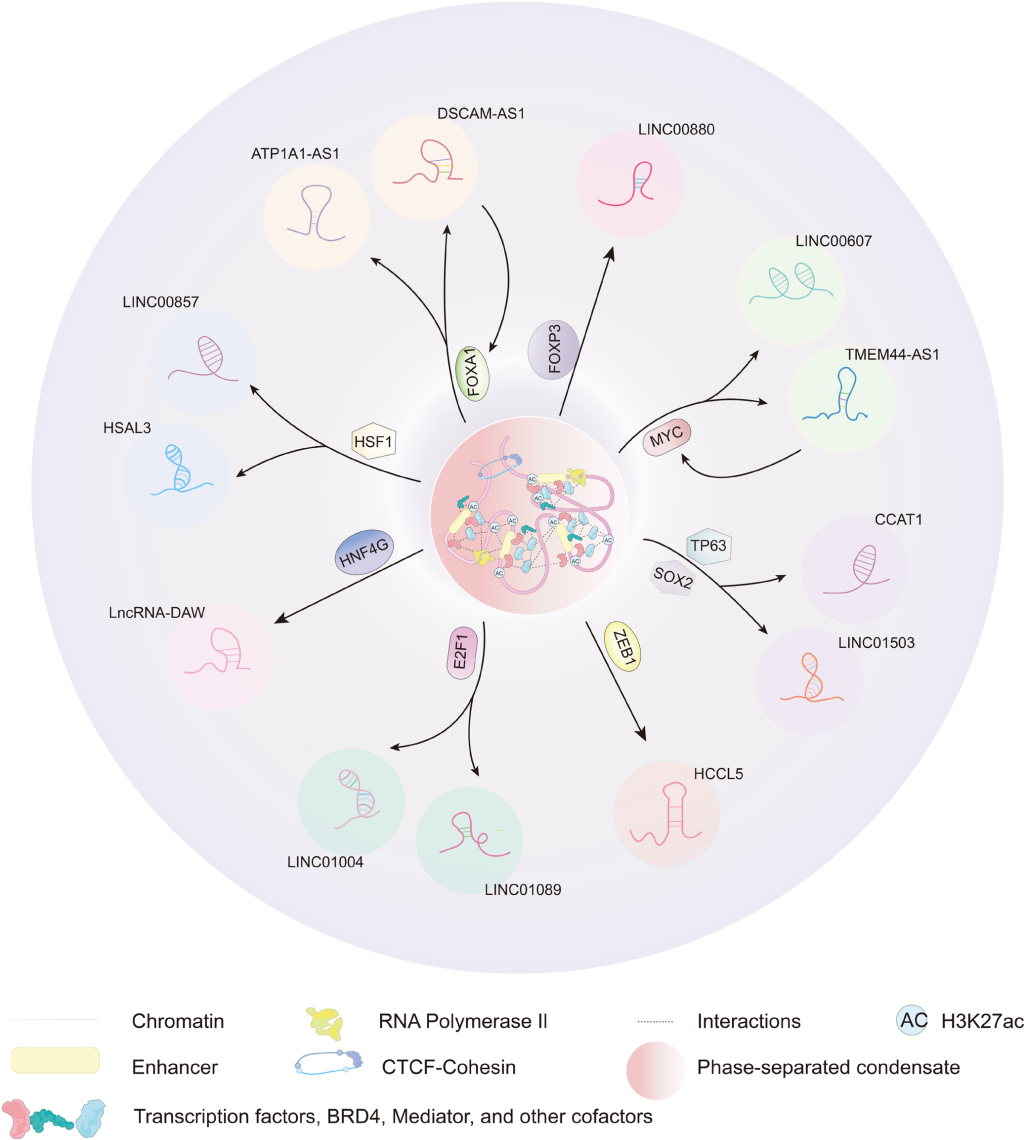
Schematic summary of several SE‐lncRNAs and corresponding TFs that are involved in the regulation of SE‐lncRNA expression and function.

Notably, the relationship between SE‐lncRNAs and TFs is not one‐to‐one; in this review, we highlight the following TFs and some of the SE‐lncRNAs they regulate (Figure 2 and Table 1).

**TABLE 1:** Transcription factors that regulate aberrant expression of SE‐lncRNA in cancer.

TF	Related SE‐lncRNA	Tumour type	Mechanism	References
HSF1	LINC00857	Colorectal cancer	Promote the transcription of LINC00857	[43]
	HSAL3	Hepatocellular carcinoma	Upregulation and transcriptional activation of HSAL3	[44]
HNF4G	LncRNA‐DAW	Hepatocellular carcinoma	Transcriptional activation of lncRNA‐DAW	[45]
E2F1	LINC01004	Hepatocellular carcinoma	Bind to LINC01004 SE to promote its expression	[46]
	LINC01089	Hepatocellular carcinoma	Bind to LINC01089 SE and promotes its transcription and expression	[47]
ZEB1	HCCL5	Hepatocellular carcinoma	Upregulation and transcriptional activation of HCCL5	[18]
TP63/SOX2	LINC01503	Oesophageal squamous cell carcinomas	Bind to LINC01503 SE to promote its expression and activate its transcription	[19]
	CCAT1	Squamous cell carcinomas	Regulation of CCAT1 expression through activation of its SE and promoter	[17]
MYC	TMEM44‐AS1	Glioma	Transcriptional activation of TMEM44‐AS1, and the formation of a positive feedback loop with it	[48]
	LINC00607	Hepatocellular carcinoma	Interact with the LINC00607 promoter and upregulate it	[49]
FOXP3	LINC00880	Lung adenocarcinoma	Occupy the promoter and SE region of LINC00880 to promote its transcription	[41]
FOXA1	ATP1A1‐AS1	Breast cancer	Bind to ATP1A1‐AS1 SE to regulate its expression	[50]
	DSCAM‐AS1	Lung malignant adenomas, breast and prostate cancer	Transcriptional activation DSCAM‐AS1 and the positive feedback loop formed with it	[51]

### 4.1 HSF1

Heat shock transcription factor 1 (HSF1) broadly influences tumour biology [52, 53, 54, 55]. HSF1 promotes cancer progression by orchestrating a range of essential cellular processes [56].

In CRC, HSF1 can activate the transcription of long intergenic nonprotein coding RNA 857 (LINC00857), which is regulated by SEs. Additionally, H3K27ac is essential for HSF1 expression. This finding suggests a positive feedback loop between the TFs HSF1 and SE that promotes the continuous activation of HSF1 and LNC00857, thus continuously activating the downstream signalling pathway [43].

The lncRNA RP13‐890H12.2 (HSAL3) is a novel SE‐lncRNA. In hepatocellular carcinoma (HCC) tissues, HSAL3 is upregulated and activated by the TF HSF1 through its SE, which, in turn, interferes with the negative regulation of NOTCH signalling [44]. Given that NOTCH signalling influences many aspects of cancer biology, HSAL3 may play a role in tumour promotion through this mechanism [57].

### 4.2 HNF4G

Hepatocyte nuclear factor 4 (HNF4) is a type of TF that binds fatty acids [58]. In the peripheral regions, enterocytes and hepatocytes rely on the HNF4 TF family, which consists of two homologues, hepatocyte nuclear factor 4 alpha (HNF4A) and hepatocyte nuclear factor 4 gamma (HNF4G), to facilitate their differentiation and function [59].

Recently, an SE‐lncRNA, lncRNA‐DAW, was identified as a biomarker for liver cancer. This lncRNA is activated by HNF4G and its associated SE, leading to abnormal transcriptional activity in HCC. LncRNA‐DAW plays a crucial role in liver cancer development by activating the expression of Wnt family member 2 (Wnt2). This activation promotes aberrant expression of the Wnt/β‐catenin signalling pathway, which is associated with tumour development [45].

### 4.3 E2F1

E2f transcription factor 1 (E2F1), a member of the E2F family of transcription factors, can control the expression of target genes by binding to their promoters at the transcriptional level [60].

E2F1 participates in regulating several SE‐lncRNAs, including LINC01004 and LINC01089 [46, 47]. One SE‐lncRNA, long intergenic nonprotein coding RNA 1089 (LINC01089), is closely linked to the progression of HCC. E2F1 binding to the LINC01089 SE significantly increases the expression of this lncRNA. Thus, the dependence of LINC01089 on its SE may be attributed to E2F1 binding, which enhances gene transcription [47].

### 4.4 ZEB1

Zinc finger E‐box‐binding protein 1 (ZEB1) is well known for its role in promoting epithelial‐to‐mesenchymal transition (EMT) and is involved in various tumorigenic activities, including cell apoptosis, chemotherapy resistance, invasion and metastasis. Additionally, ZEB1 can induce stem‐cell properties [61, 62, 63, 64].

The SE‐lncRNA HCCL5 promotes EMT in HCC cells by upregulating the expression of EMT‐related TFs. HCCL5 is transcriptionally regulated by ZEB1. ZEB1 interacts with both the SE and promoter regions of HCCL5 to increase its transcription during both steady‐state conditions and EMT processes [18].

### 4.5 TP63/SOX2

Recent studies have demonstrated that the overexpression of SRY‐box transcription factor 2 (SOX2) and TP63 promotes proliferation and tumorigenesis in squamous cells, highlighting their carcinogenic roles [65, 66, 67].

TP63 and SOX2 are implicated in cancer progression by their ability to modify the levels of SE‐lncRNAs, such as LINC01503 and CCAT1 [17, 19]. CCAT1 is an SE‐lncRNA that is strongly expressed in ESCC. In nearly all squamous cell carcinoma (SCC) cell lines, the promoter and SE regions of CCAT1 are co‐occupied by TP63 and SOX2. These findings suggest that CCAT1 is a downstream target of TP63 and SOX2 in SCC [17]. Furthermore, CCAT1 interacts with TP63 and binds to EGFR super‐enhancers along with SOX2, increasing the activity of EGFR. This interaction activates the MEK/ERK1/2 and PI3K/AKT signalling pathways, driving SCC cell proliferation and survival [68, 69].

### 4.6 MYC

The TF MYC is crucial for regulating numerous cellular processes and is closely associated with cancer development [48, 70, 71]. MYC uniquely influences the regulation of SE‐lncRNAs and is involved in the transcriptional control of several SE‐lncRNAs, including TMEM44‐AS1 and LINC00607 [48, 49].

Recent studies revealed that TMEM44‐AS1, a novel SE‐lncRNA, is dysregulated in glioma tissues. The presence of TMEM44‐AS1 enhances glioma cell proliferation, colony formation, migration and invasion. During glioma progression, TMEM44‐AS1 binds directly to SerpinB3 and sequentially activates MYC and the EGR1/IL‐6 signalling pathway. Conversely, MYC binds to the TMEM44‐AS1 SE, promoting its glioma‐specific transcriptional activation. This interaction creates a positive feedback loop involving TMEM44‐AS1 and MYC, highlighting the importance of the TMEM44‐AS1‐MYC axis in glioma progression [48].

### 4.7 FOXP3

The overexpression of forkhead box protein P3 (FOXP3) significantly promotes cellular proliferation, migration and invasion, indicating its potential as a carcinogen [72, 73, 74, 75, 76].

The SE‐lncRNA long intergenic nonprotein coding RNA 880 (LINC00880) promotes cell growth, invasion and metastasis in lung adenocarcinoma (LUAD) by forming a complex with CDK1 and PRDX1. This complex regulates the PTEN‐AKT pathway, promoting malignancy. The TF FOXP3 simultaneously binds to the promoter and SE regions of LINC00880, resulting in the upregulation of its transcription. These findings suggest that FOXP3 contributes to cancer progression by regulating LINC00880 levels [41].

### 4.8 FOXA1

The FOXA1 protein belongs to a unique category of TFs called pioneer factors. FOXA1 plays a crucial role in cancer development [51, 77, 78, 79].

FOXA1 regulates several SE‐lncRNAs, including ATP1A1‐AS1 and DSCAM‐AS1 [50, 51]. DSCAM‐AS1, an SE‐lncRNA, is abnormally expressed in lung adenomas, as well as in breast and prostate cancers, and is directly targeted by FOXA1 [51, 80, 81]. FOXA1 binds directly to the promoter of DSCAM‐AS1, modulating its expression in cancer cells. Notably, DSCAM‐AS1 also regulates FOXA1 expression at the transcriptional level. This bidirectional regulation highlights that SE‐lncRNAs can influence TF expression and contribute to the formation of CRC [51].

In conclusion, dysregulation of the expression of SE‐lncRNAs and their associated TFs is frequently observed in cancer cells and tissues. This dysregulation often results in the activation or inhibition of downstream targets, which significantly contributes to cancer progression and affects patient outcomes. Therefore, SE‐lncRNAs and their associated TFs have significant potential as diagnostic biomarkers and therapeutic targets in cancer treatment.

## 5 The Mechanisms Underlying the Complexity of SE‐lncRNAs

**FIGURE 3:**
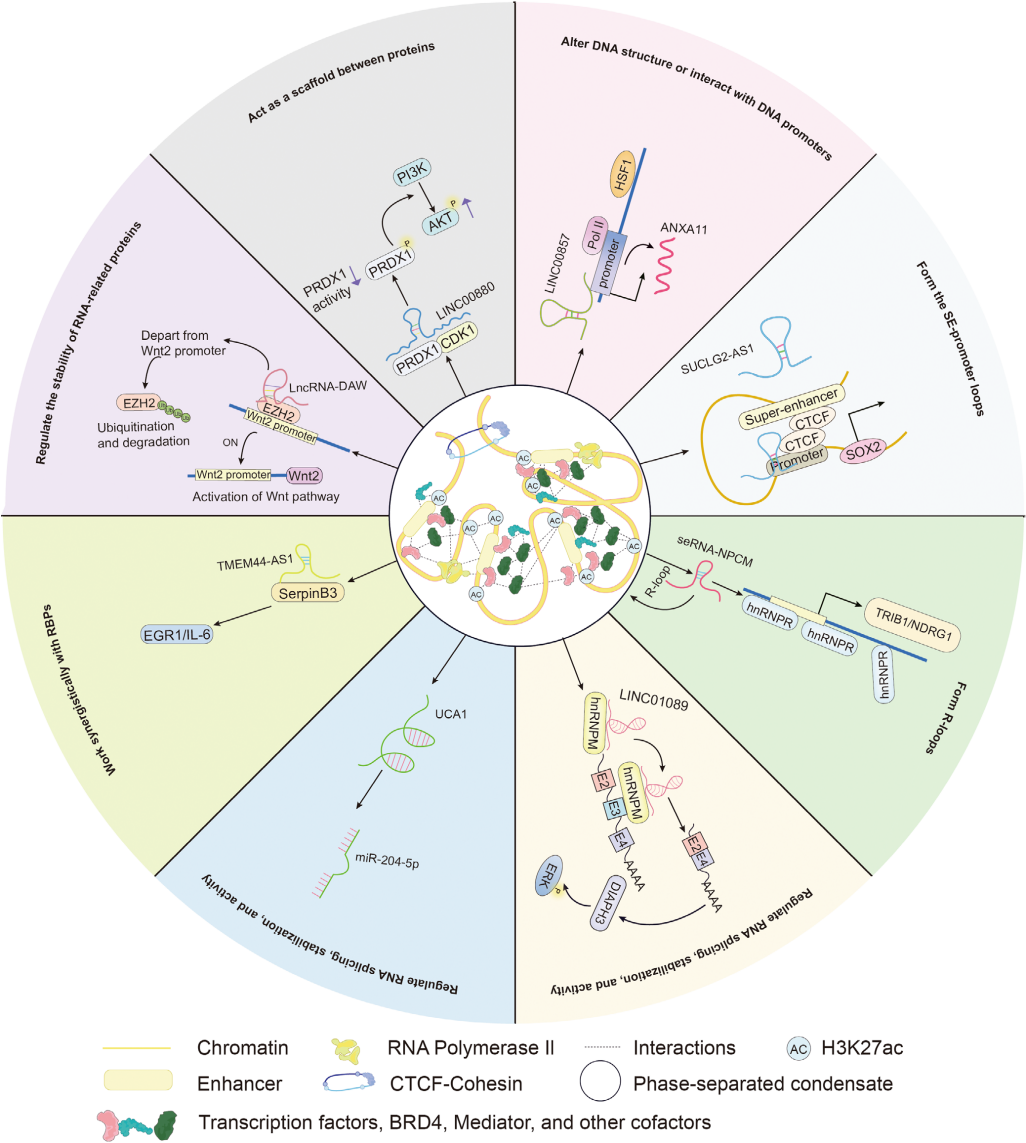
Schematic demonstration of the potential regulatory mechanisms by which SE‐lncRNAs participate in cancer progression.

Increasing evidence suggests that lncRNAs are involved in almost the entire life cycle of cells through different mechanisms and play important roles in many critical biological processes. Therefore, exploring the relevant interactions of lncRNAs is particularly important for the mechanistic understanding, treatment, prognosis and prevention of human cancers at the lncRNA level [82]. Furthermore, lncRNAs have the potential to serve as reliable diagnostic biomarkers and therapeutic targets for cancer [83]. These insights highlight the importance of exploring the roles of super‐enhancer–associated lncRNAs (SE‐lncRNAs) in cancer to deepen our understanding of cancer cell metabolism and progression. This review examines the mechanisms through which SE‐lncRNAs contribute to cancer progression at the DNA, mRNA and protein levels (Figure 3 and Table 2).

**TABLE 2:** Molecular mechanisms and functions of SE‐lncRNAs involved in carcinogenesis.

Mechanisms	SE‐lncRNA	Subcellular localisation	Effects on cancer development	Biological function	References
Alter DNA structure or interact with DNA promoters	LINC00857	Nucleus	Oncogene	Cell proliferation	[43]
Form the SE‐promoter loops	SUCLG2‐AS1	Nucleus	Oncogene	Promote cell invasion, metastasis and radio‐resistance	[21]
Form R‐loops	seRNA‐NPCM	Nucleus and cytoplasm	Oncogene	Promote cell metastasis, and invasion	[84]
Regulate RNA splicing, stabilisation, and activity	LINC01089	Nucleus	Oncogene	EMT, migration, invasion and metastasis	[47]
	UCA1	Cytoplasm	Oncogene	ECM remodelling, cancer angiogenesis, cell proliferation, migration, invasion and apoptosis	[85, 86, 87]
Work synergistically with RBPs	TMEM44‐AS1	Nucleus and cytoplasm	Oncogene	Cell proliferation, colony formation, migration and invasion	[48]
	DSCAM‐AS1	/	Oncogene	Cell proliferation and colony growth	[51]
Regulate the stability of RNA‐related proteins	LncRNA‐DAW	/	Oncogene	Cell growth, metastasis and invasion	[45]
	AC005592.2	Nucleus	Oncogene	Cell proliferation, invasion, migration and induces apoptosis	[88]
	RP11‐569A11.1	Nucleus	Antioncogene	Inhibits the proliferation and metastasis	[23]
	LINC00945	/	Oncogene	Cell proliferation, EMT, migration, invasion and immune response	[89]
	HCCL5	Cytoplasm	Oncogene	Modulation of EMT	[18]
Act as a scaffold between proteins	LINC00880	/	Oncogene	Cell migration. invasion and cell proliferation	[41]

### 5.1 SE‐lncRNAs Alter DNA Structure or Interact With DNA Promoters in Cancer

The early stage of tumorigenesis often involves the accumulation of genetic mutations, including chromosome translocations. These genetic alterations can disrupt normal cellular processes and contribute to the uncontrolled proliferation of malignant tumours [90].

Aberrant SE‐lncRNA synthesis can indeed play a significant role in tumorigenesis. For example, the transcription of SE‐lncRNAs can lead to activation‐induced cytidine deaminase (AID) mistargeting, which contributes to genome instability and malignancy [91]. Specifically, certain SE‐lncRNAs located within the BCL6 gene breakpoint region align with the BCL6 translocation region [92, 93]. These SE‐lncRNAs are transcribed in the opposite direction from the BCL6 gene, generating single‐stranded DNA. This single‐stranded DNA is then subject to mutations by AID, which can cause double‐strand breaks and chromosome translocations. Studies have shown that AID specifically deaminates cytidine targets within this breakpoint region, thereby promoting DNA double‐strand breaks and subsequent chromosomal rearrangements [92, 93, 94, 95]. These findings suggest that SE‐lncRNAs have the potential to alter DNA structure and may thereby affect gene expression and lead to cancer progression.

In addition to inducing changes in DNA structure, SE‐lncRNAs can interact with DNA promoters to influence downstream processes and ultimately contribute to the regulation of cancer progression. A recent study revealed that an SE‐lncRNA called LINC00857 is associated with CRC progression [43, 96]. LINC00857 specifically targets the promoter region of annexin A11 (ANXA11), leading to increased transcription of this gene. Silencing LINC00857 significantly reduces the presence of the transcriptional activator RNA polymerase II (RNA Pol II) at the ANXA11 promoter [43]. Consequently, by affecting the ANXA11 promoter, LINC00857 promotes the activation of the HSF1/LINC00857/ANXA11 signalling axis, which enhances the proliferation and spread of cancer cells [43].

### 5.2 SE‐lncRNAs Indirectly Form SE–Promoter Loops in Cancer

SE‐lncRNAs play crucial roles in regulating gene expression through their interactions with enhancers and promoters. They help initiate and maintain chromatin loops between these elements, thereby influencing transcriptional activity [35, 97].

CTCF, a multifunctional TF, plays a significant role in this process [98]. SE‐lncRNA SUCLG2‐AS1 recruits CTCF, which facilitates the formation of chromatin loops in the SE region of SOX2 with the promoter region. This remote interaction increases the transcriptional activation of SOX2 and promotes invasion and metastasis of nasopharyngeal carcinoma (NPC) [21].

### 5.3 SE‐lncRNAs are Involved in Forming R‐Loops in Cancer

R‐loops, which are RNA–DNA hybrids, have important roles in cancer‐associated effects such as DNA damage, hyperrecombination and genome instability [84]. Changes in the frequency, stability or genetic positioning of R‐loops, such as the triggering of oncogenes or the loss of tumour‐suppressor genes, are associated with the progression of cancer [99]. SE‐lncRNAs can form R‐loops with SEs in cancer, thereby interfering with DNA replication and thus inducing chromosomal rearrangements and genomic instability [100].

The seRNA‐NPCM is a specific type of SE‐lncRNA involved in the metastasis of NPC. It is linked to the regulation of N‐myc downstream‐regulated gene 1 (NDRG1), which has been found to promote NPC metastasis. The mechanism involves the overexpression of seRNA‐NPCM, which leads to increased R‐loop formation via interaction with the SE region at its 3′ end. This R‐loop facilitates the formation of chromatin loops between the SE and the NDRG1 promoter, thereby enhancing the transcription of NDRG1. In this process, heterogeneous nuclear ribonucleoprotein R (hnRNPR) is the protein partner of seRNA‐NPCM, which binds seRNA‐NPCM to the promoters of TRIB1 and NDRG1. As a result, this process significantly contributes to the metastatic potential of NPC cells [84].

However, how super‐enhancer regions produce high levels of SE‐lncRNAs without significantly limiting R‐loop formation remains an unresolved question. Further research is needed to understand the balance between SE‐lncRNA production and R‐loop dynamics in cancer cells.

### 5.4 SE‐lncRNAs Regulate RNA Splicing, Stabilisation and Activity in Cancer

RNA plays crucial roles in gene expression, whether as protein‐coding RNAs (mRNAs) or as noncoding RNAs involved in transcription (e.g., lncRNAs), splicing and translation (e.g., miRNAs) [101, 102, 103, 104, 105, 106]. Recent evidence has shown that RNA processing is systematically altered in cancer, underscoring the impact of RNA on tumorigenesis, growth and progression [106, 107, 108, 109, 110]. These alterations in RNA processing are commonly observed in cancer and can drive tumorigenesis [106]. SE‐lncRNAs, a specialised class of lncRNAs, may also influence cancer progression by modulating RNA processing.

Long intergenic nonprotein coding RNA 1089 (LINC01089) is specifically expressed in HCC cells. LINC01089 increases the splicing of DIAPH3 mRNA, which is controlled by hnRNPM, and reduces the incorporation of m6A‐modified exon 3 into DIAPH3 mRNA. This leads to decreased DIAPH3 mRNA stability and reduced DIAPH3 expression, which, in turn, increases ERK signalling activation and promotes HCC metastasis and progression [47].

Increasing evidence suggests that ceRNAs play crucial roles in the development of various cancers by acting as sponges for miRNAs and modulating the expression of target genes [85, 111, 112, 113]. In CRC, the SE‐lncRNA urothelial carcinoma associated 1 (UCA1) functions as a ceRNA for miR‐204‐5p. UCA1 binds to miR‐204‐5p and suppresses its activity, leading to increased expression of miR‐204‐5p target genes such as CREB1, BCL2 and RAB22A in CRC cells [85, 86, 87]. These findings suggest that SE‐lncRNAs can significantly influence posttranslational modifications and cancer development by functioning as molecular sponges or ceRNAs [31].

### 5.5 SE‐lncRNAs Work Synergistically With RBPs in Cancer

Accumulating evidence indicates that cellular processes such as cell proliferation, apoptosis and cancer metastasis are regulated by lncRNA‐RBP (RNA‐binding protein) interactions [114]. LncRNAs perform their cellular functions by forming macromolecular complexes with proteins [114, 115]. The accurate prediction of lncRNA–protein interactions (LPIs) is essential for elucidating lncRNA function and pathogenesis. Several methods have been developed to efficiently and accurately predict LPIs, including the use of the FMSRT‐LPI model to accurately identify potential LPIs [116, 117, 118, 119]. Disruptions in the lncRNA‐RBP interaction network are closely linked to cancer development and progression.

The aberrant expression of the novel SE‐lncRNA TMEM44‐AS1 in glioma tissues is associated with malignant progression and poor survival outcomes in glioma patients. This is due to the direct interaction between TMEM44‐AS1 and SerpinB3, which facilitates the binding of SerpinB3 to downstream targets, leading to the activation of the EGR1/IL‐6 signalling pathway and the subsequent promotion of cancer progression [48]. Similarly, DSCAM‐AS1, another SE‐lncRNA, is specifically expressed in lung, breast and prostate adenocarcinomas. In these cancers, DSCAM‐AS1 modulates the expression of FOXA1 and ERα by interacting with YBX1, influencing YBX1 recruitment to the promoter regions of FOXA1 and Erα and thereby promoting tumour progression [51].

### 5.6 SE‐lncRNAs Regulate the Stability of RNA‐Related Proteins in Cancer

LncRNAs, including SE‐lncRNAs, play crucial roles in cancer development through various mechanisms, such as regulating protein stability and posttranslational modifications such as phosphorylation [120].

Recent studies have identified a liver‐specific SE‐lncRNA, lncRNA‐DAW (distant activator of Wnt2), which destabilises the enhancer of zeste homologous 2 (EZH2) protein. It does so by altering the ubiquitination and phosphorylation of EZH2, leading to decreased stability and reduced total EZH2 protein levels [45]. This decrease in EZH2 contributes to the amplification of Wnt2, ultimately promoting cancer progression [45, 121, 122, 123, 124].

### 5.7 SE‐lncRNAs Act as Scaffolds Between Proteins in Cancer

The SE‐lncRNA LINC00880 is a relatively specific regulator of LUAD [41]. It assists in the connection of CDK1 and PRDX1, serving as a structural support for the formation of a ternary complex. This complex modulates the level of phosphorylated PRDX1, resulting in the activation of PI3K/AKT. Ultimately, this activation promotes the development of malignant tumours [41].

In summary, SE‐lncRNAs play pivotal roles in regulating cancer development. They influence cancer progression through interactions with chromatin—by facilitating loop formation and modulating chromatin stability—or by interacting with RBPs to regulate downstream signalling pathways. These findings suggest that SE‐lncRNAs act as molecular scaffolds, coordinating various factors at specific gene expression sites to ensure precise regulation, thereby impacting cancer development and progression.

## 6 Potential Functions of SE‐lncRNAs

**FIGURE 4:**
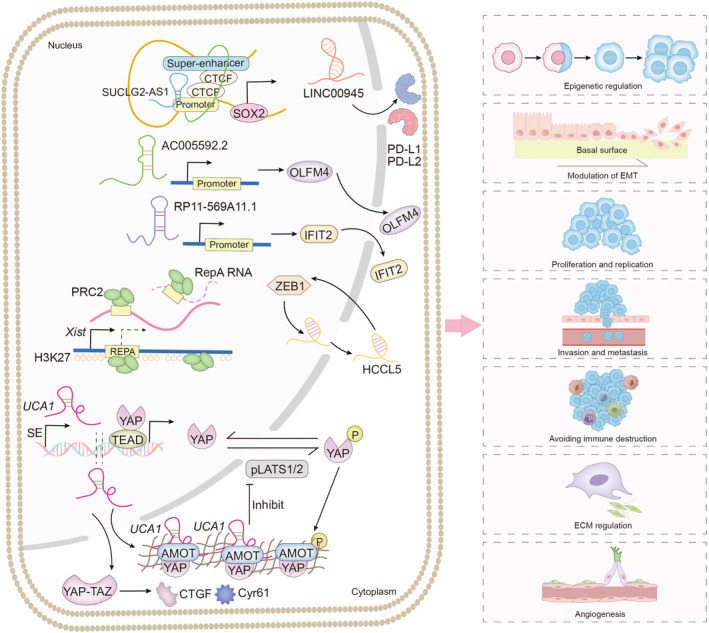
Complex functions and signalling of SE‐lncRNAs involved in cancer progression.

Recent research has highlighted the active role of SE‐lncRNAs in a wide range of pathological mechanisms in human cancers. These roles are facilitated through their involvement in super‐enhancer activation and interactions with various proteins and molecules [23]. This review focuses on the key functions of SE‐lncRNAs in the cancer process (Figures 1C and 4 and Table 2).

### 6.1 Roles in the Modulation of EMT in Cancer

EMT is a key process in the progression of malignant epithelial tumours that leads to the development of more aggressive and metastatic tumour subtypes. EMT is crucial in promoting tumour growth, increasing the population of cancer stem cells and facilitating tumour metastasis [125].

The SE‐lncRNA HCCL5, which is associated with liver cancer, shows increased expression in classical EMT models induced by TGF‐β1. ZEB1 interacts with the SE and promoter regions of HCCL5 to increase its transcription in both homeostasis and EMT. HCCL5 enhances the EMT phenotype by increasing the levels of key transcription factors, including Snail, ZEB1 and Twist1 [18].

### 6.2 Roles in Vital Activities of Cancer Cells

Cancer growth is characterised by an imbalance in which proliferating tumour cells outnumber those that undergo apoptosis, disrupting the equilibrium between cell proliferation and cell death [126]. This fundamental imbalance is closely associated with alterations in cancer cell metabolism, which can either result from or contribute to tumour progression [127]. SE‐lncRNAs play a significant role in this process by modulating various cellular activities, influencing both the metabolic state of cancer cells and their overall behaviour.

SE‐lncRNA AC005592.2 is located primarily in the nucleus and is significantly upregulated in CRC tissues and cells. This CRC‐specific marker promotes cancer cell proliferation and metastasis by positively regulating the expression of its downstream gene, olfactomedin 4 (OLFM4) [88]. Elevated levels of AC005592.2, thus, contribute to cancer progression in CRC. However, SE‐lncRNAs can have dual roles in cancer. For example, the novel SE‐lncRNA RP11‐569A11.1, which is implicated in CRC progression, functions as a tumour suppressor through its interaction with IFIT2. RP11‐569A11.1 is downregulated in CRC tissues, leading to reduced expression of IFIT2. This reduction inhibits CRC cell proliferation and metastasis while promoting the apoptosis of tumour cells, indicating that RP11‐569A11.1 acts to suppress tumours in CRC [23].

### 6.3 Roles in the Immune Response in Cancer

According to the concept of immunosurveillance, the immune system remains vigilant, continuously monitoring cells and tissues to detect and eliminate early cancer cells [128]. Among various forms of immunotherapy, immune checkpoint blockade (ICB) has been extensively researched [129]. Nevertheless, tumour tissue generally does not possess immunological checkpoints and persists in its proliferation. Recent research has shown that SE‐related oncogenes influence the regulation of both inhibitory and stimulatory immune checkpoints [31].

A study developed a model using SE‐lncRNAs as biomarkers to evaluate the clinical prognosis of glioma patients. This research revealed that certain SE‐lncRNAs, such as LINC00945, can affect the infiltration and abundance of immune cells, including natural killer (NK) cells, T follicular helper (Tfh) cells and Th1 cells. Additionally, the expression levels of these SE‐lncRNAs may positively correlate with the expression levels of associated immune checkpoint genes [89]. Furthermore, research has identified an SE in breast cancer that elevates the levels of programmed death‐ligand 1 (PD‐L1) and programmed death‐ligand 2 (PD‐L2), thereby helping tumours evade the immune system [130].

These findings indicate that SE‐lncRNAs have the potential to modulate cancer progression through their impact on immune cell function.

### 6.4 Roles in ECM Remodelling in Cancer

The extracellular matrix (ECM) plays a critical role in various cellular activities that advance cancer and serves as a reservoir for substances that regulate tumour cell behaviour [131].

Recent research has highlighted the role of the SE‐lncRNA UCA1 in activating YAP, revealing that dysregulated activation of the YAP/TAZ axis is observed in the tumour microenvironment of several cancers, including lung and breast cancer [85, 132]. YAP/TAZ activation significantly enhances contractile activity and increases the expression of connective tissue growth factor (CTGF) and cysteine‐rich angiogenic inducer 61 (Cyr61), promoting ECM protein deposition [133]. Notably, YAP can sustain its own activity by inducing the expression of myosin regulatory light chain 9 (MYL9) and increasing actin contractility, leading to ‘inside‐out’ ECM sclerosis [125]. Additionally, previous studies have identified several SE‐lncRNAs in hepatic stellate cells that regulate ECM stiffness in a unidirectional manner [134, 135].

### 6.5 Roles in Cancer Angiogenesis

Angiogenesis promotes cancer progression by facilitating the provision of nutrients and energy, making it a common target for cancer therapy [136]. SE‐lncRNAs play a role in modulating cancer angiogenesis by activating various signalling pathways [135].

The Hippo–YAP signalling pathway, which is activated by the SE‐lncRNA UCA1, has been shown to promote angiogenesis in several cancer types, including pancreatic ductal adenocarcinoma (PDAC) and EOC, through multiple mechanisms. Specifically, UCA1 enhances the expression of Ang2, VE‐cadherin and α‐smooth muscle actin (α‐SMA) [87, 135, 137, 138]. Mechanistically, UCA1 in the cytoplasm interacts with AMOT to promote the interaction between AMOT and YAP. This binding prevents interaction between pLATS1/2 and YAP as well as YAP phosphorylation. Consequently, this promotes the nuclear translocation of YAP and its interaction with TEAD to activate the expression of oncogene tags [87].

A comprehensive analysis revealed that SE‐lncRNAs play dualistic roles in cancer development, acting both as oncogenes that promote tumour progression and as tumour suppressors that inhibit cancer progression. Both upregulated and downregulated SE‐lncRNAs influence cancer cell behaviours, including proliferation, invasion, metastasis and apoptosis. However, the precise mechanisms underlying these effects remain unclear and may involve distinct targets or signalling pathways. Additionally, the expression of SE‐lncRNAs appears to be cell‐type specific, indicating their potential as biomarkers for identifying and classifying different tumour subtypes across various systems [139].

## 7 Concluding Remarks

In recent years, significant advances have enhanced our understanding of super‐enhancer–associated long noncoding RNAs (SE‐lncRNAs), providing deeper insight into their characteristics and multifunctional roles.

A very high number of lncRNAs that play roles in various cancers have been reported, and clarifying their specific mechanisms and then targeting their associated SEs is an important research direction for the treatment of tumours [17, 97]. SE‐lncRNAs may serve as novel targets for tumour therapeutics. Xu et al. suggested that SE‐lncRNAs might provide a breakthrough in immunotherapy for solid tumours [140, 141]. A diverse array of preclinical studies indicates that SE inhibitors, such as the BRD4 inhibitor JQ1, exhibit significant potential in repressing seRNA transcription and impeding cancer proliferation [142, 143, 144]. Notably, JQ1 has been shown to preferentially repress SE‐lncRNA transcription compared with other lncRNAs [44]. However, it remains unclear whether all SE inhibitors can regulate SE‐lncRNAs. If they can, they could offer new avenues for anticancer strategies.

The impact of SE‐lncRNAs on intricate physiological processes and the development of cancer is a topic of considerable importance. In contrast to previous studies that have generally focused on the involvement of SE‐lncRNAs in cancer progression, the present review provides an in‐depth exploration of the various mechanisms by which SE‐lncRNAs participate in the regulation of tumour progression, as well as the biological functions of SE‐lncRNAs in this process. However, there are limitations: specific SE‐lncRNAs exhibit different expression levels across various tumour types and influence diverse tumour phenotypes. While tissue specificity may explain some of this variability, the precise mechanisms are not fully understood.

Furthermore, extensive research is needed to better understand the relationship between SE‐lncRNAs and cancer. Given their unique attributes, SE‐lncRNAs are expected to play an increasingly important role in personalised medicine.

## Author Contributions

**Yuxin Bao:** formal analysis (equal), methodology (equal), supervision (equal), writing – original draft (equal). **Songling Teng:** methodology (equal), supervision (equal), validation (equal), visualization (equal). **Hanjie Zhai:** investigation (equal), methodology (equal), validation (equal), visualization (equal). **Yuanzhuang Zhang:** formal analysis (equal), methodology (equal), software (equal), visualization (equal). **Yeqiu Xu:** investigation (equal), methodology (equal), software (equal), validation (equal). **Chenghao Li:** formal analysis (equal), investigation (equal), supervision (equal). **Zhenjun Chen:** formal analysis (equal), methodology (equal), resources (equal), supervision (equal). **Fu Ren:** data curation (equal), formal analysis (equal), investigation (equal), validation (equal), visualization (equal). **Yong Wang:** conceptualization (lead), data curation (equal), funding acquisition (lead), investigation (equal), resources (equal), writing – review and editing (equal).

## Conflicts of Interest

The authors declare no conflicts of interest.

## Data Availability

The authors have nothing to report.

## References

[1] K. E. de Visser and J. A. Joyce , “The Evolving Tumor Microenvironment: From Cancer Initiation to Metastatic Outgrowth,” Cancer Cell 41, no. 3 (2023): 374–403. 36917948 10.1016/j.ccell.2023.02.016

[2] R. Esposito , N. Bosch , A. Lanzós , T. Polidori , C. Pulido‐Quetglas , and R. Johnson , “Hacking the Cancer Genome: Profiling Therapeutically Actionable Long Non‐Coding RNAs Using CRISPR‐Cas9 Screening,” Cancer Cell 35, no. 4 (2019): 545–557. 30827888 10.1016/j.ccell.2019.01.019

[3] L. Lin , Z. Li , L. Yan , Y. Liu , H. Yang , and H. Li , “Global, Regional, and National Cancer Incidence and Death for 29 Cancer Groups in 2019 and Trends Analysis of the Global Cancer Burden, 1990–2019,” Journal of Hematology & Oncology 14, no. 1 (2021): 197. 34809683 10.1186/s13045-021-01213-z PMC8607714

[4] S. Djebali , C. A. Davis , A. Merkel , et al., “Landscape of Transcription in Human Cells,” Nature 489, no. 7414 (2012): 101–108. 22955620 10.1038/nature11233 PMC3684276

[5] J. Ferrer and N. Dimitrova , “Transcription Regulation by Long Non‐Coding RNAs: Mechanisms and Disease Relevance,” Nature Reviews Molecular Cell Biology 25, no. 5 (2024): 396–415. 38242953 10.1038/s41580-023-00694-9 PMC11045326

[6] J. S. Mattick , P. P. Amaral , P. Carninci , et al., “Long Non‐Coding RNAs: Definitions, Functions, Challenges and Recommendations,” Nature Reviews Molecular Cell Biology 24, no. 6 (2023): 430–447. 36596869 10.1038/s41580-022-00566-8 PMC10213152

[7] D. M. Anderson , K. M. Anderson , C. L. Chang , et al., “A Micropeptide Encoded by a Putative Long Noncoding RNA Regulates Muscle Performance,” Cell 160, no. 4 (2015): 595–606. 25640239 10.1016/j.cell.2015.01.009 PMC4356254

[8] L. Statello , C. J. Guo , and L. L. Chen , “Gene Regulation by Long Non‐Coding RNAs and Its Biological Functions,” Nature Reviews Molecular Cell Biology 22, no. 2 (2021): 96–118. 33353982 10.1038/s41580-020-00315-9 PMC7754182

[9] B. Soibam , “Super‐lncRNAs: Identification of lncRNAs That Target Super‐Enhancers via RNA:DNA:DNA Triplex Formation,” RNA 23, no. 11 (2017): 1729–1742. 28839111 10.1261/rna.061317.117 PMC5648039

[10] W. A. Whyte , D. A. Orlando , D. Hnisz , et al., “Master Transcription Factors and Mediator Establish Super‐Enhancers at Key Cell Identity Genes,” Cell 153, no. 2 (2013): 307–319. 23582322 10.1016/j.cell.2013.03.035 PMC3653129

[11] E. P. Nora , B. R. Lajoie , E. G. Schulz , et al., “Spatial Partitioning of the Regulatory Landscape of the X‐Inactivation Centre,” Nature 485, no. 7398 (2012): 381–385. 22495304 10.1038/nature11049 PMC3555144

[12] S. Dębek and P. Juszczyński , “Super Enhancers as Master Gene Regulators in the Pathogenesis of Hematologic Malignancies,” Biochimica et Biophysica Acta‐Reviews on Cancer 1877, no. 2 (2022): 188697. 35150791 10.1016/j.bbcan.2022.188697

[13] T. Henriques , B. S. Scruggs , M. O. Inouye , et al., “Widespread Transcriptional Pausing and Elongation Control at Enhancers,” Genes & Development 32, no. 1 (2018): 26–41. 29378787 10.1101/gad.309351.117 PMC5828392

[14] R. Andersson , C. Gebhard , I. Miguel‐Escalada , et al., “An Atlas of Active Enhancers Across Human Cell Types and Tissues,” Nature 507, no. 7493 (2014): 455–461. 24670763 10.1038/nature12787 PMC5215096

[15] C. C. Hon , J. A. Ramilowski , J. Harshbarger , et al., “An Atlas of Human Long Non‐Coding RNAs With Accurate 5′ Ends,” Nature 543, no. 7644 (2017): 199–204. 28241135 10.1038/nature21374 PMC6857182

[16] K. Schaukowitch , J. Y. Joo , X. Liu , J. K. Watts , C. Martinez , and T. K. Kim , “Enhancer RNA Facilitates NELF Release From Immediate Early Genes,” Molecular Cell 56, no. 1 (2014): 29–42. 25263592 10.1016/j.molcel.2014.08.023 PMC4186258

[17] Y. Jiang , Y. Y. Jiang , J. J. Xie , et al., “Co‐Activation of Super‐Enhancer‐Driven CCAT1 by TP63 and SOX2 Promotes Squamous Cancer Progression,” Nature Communications 9, no. 1 (2018): 3619. 10.1038/s41467-018-06081-9 PMC6127298 30190462

[18] L. Peng , B. Jiang , X. Yuan , et al., “Super‐Enhancer‐Associated Long Noncoding RNA HCCL5 Is Activated by ZEB1 and Promotes the Malignancy of Hepatocellular Carcinoma,” Cancer Research 79, no. 3 (2019): 572–584. 30482773 10.1158/0008-5472.CAN-18-0367

[19] J. J. Xie , Y. Y. Jiang , Y. Jiang , et al., “Super‐Enhancer‐Driven Long Non‐Coding RNA LINC01503, Regulated by TP63, Is Over‐Expressed and Oncogenic in Squamous Cell Carcinoma,” Gastroenterology 154, no. 8 (2018): 2137–2151.e1. 29454790 10.1053/j.gastro.2018.02.018

[20] Z. W. Guo , C. Xie , K. Li , et al., “SELER: A Database of Super‐Enhancer‐Associated lncRNA‐Directed Transcriptional Regulation in Human Cancers,” Database: The Journal of Biological Databases and Curation 2019 (2019): baz027. 30806704 10.1093/database/baz027 PMC6390648

[21] X. Hu , J. Wu , Y. Feng , et al., “METTL3‐Stabilized Super Enhancers‐lncRNA SUCLG2‐AS1 Mediates the Formation of a Long‐Range Chromatin Loop Between Enhancers and Promoters of SOX2 in Metastasis and Radiosensitivity of Nasopharyngeal Carcinoma,” Clinical and Translational Medicine 13, no. 9 (2023): e1361. 37658588 10.1002/ctm2.1361 PMC10474317

[22] T. Zhang , W. Xia , X. Song , et al., “Super‐Enhancer Hijacking LINC01977 Promotes Malignancy of Early‐Stage Lung Adenocarcinoma Addicted to the Canonical TGF‐β/SMAD3 Pathway,” Journal of Hematology & Oncology 15, no. 1 (2022): 114. 35982471 10.1186/s13045-022-01331-2 PMC9389757

[23] H. Chen , J. Zheng , L. Yan , X. Zhou , P. Jiang , and F. Yan , “Super‐Enhancer‐Associated Long Noncoding RNA RP11‐569A11.1 Inhibited Cell Progression and Metastasis by Regulating IFIT2 in Colorectal Cancer,” Journal of Clinical Laboratory Analysis 35, no. 6 (2021): e23780. 33942366 10.1002/jcla.23780 PMC8183909

[24] J. R. Alvarez‐Dominguez , M. Knoll , A. A. Gromatzky , and H. F. Lodish , “The Super‐Enhancer‐Derived alncRNA‐EC7/Bloodlinc Potentiates Red Blood Cell Development in Trans,” Cell Reports 19, no. 12 (2017): 2503–2514. 28636939 10.1016/j.celrep.2017.05.082 PMC6013260

[25] K. K. H. So , Y. Huang , S. Zhang , et al., “seRNA PAM Controls Skeletal Muscle Satellite Cell Proliferation and Aging Through Trans Regulation of Timp2 Expression Synergistically With Ddx5,” Aging Cell 21, no. 8 (2022): e13673. 35851988 10.1111/acel.13673 PMC9381903

[26] P. Yan , J. Y. Lu , J. Niu , et al., “LncRNA Platr22 Promotes Super‐Enhancer Activity and Stem Cell Pluripotency,” Journal of Molecular Cell Biology 13, no. 4 (2021): 295–313. 33049031 10.1093/jmcb/mjaa056 PMC8339366

[27] A. S. Ropri , R. S. DeVaux , J. Eng , S. V. Chittur , and J. I. Herschkowitz , “Cis‐Acting Super‐Enhancer lncRNAs as Biomarkers to Early‐Stage Breast Cancer,” Breast Cancer Research 23, no. 1 (2021): 101. 34717732 10.1186/s13058-021-01479-8 PMC8557595

[28] V. Sartorelli and S. M. Lauberth , “Enhancer RNAs Are an Important Regulatory Layer of the Epigenome,” Nature Structural & Molecular Biology 27, no. 6 (2020): 521–528. 10.1038/s41594-020-0446-0 PMC7343394 32514177

[29] J. Carlevaro‐Fita and R. Johnson , “Global Positioning System: Understanding Long Noncoding RNAs Through Subcellular Localization,” Molecular Cell 73, no. 5 (2019): 869–883. 30849394 10.1016/j.molcel.2019.02.008

[30] M. C. Bridges , A. C. Daulagala , and A. Kourtidis , “LNCcation: lncRNA Localization and Function,” Journal of Cell Biology 220, no. 2 (2021): e202009045. 10.1083/jcb.202009045 PMC7816648 33464299

[31] M. Wang , Q. Y. Chen , S. J. Wang , et al., “Super‐Enhancers Complexes Zoom in Transcription in Cancer,” Journal of Experimental & Clinical Cancer Research 42, no. 1 (2023): 183. 37501079 10.1186/s13046-023-02763-5 PMC10375641

[32] P. R. Moreau , T. Örd , N. L. Downes , et al., “Transcriptional Profiling of Hypoxia‐Regulated Non‐Coding RNAs in Human Primary Endothelial Cells,” Frontiers in Cardiovascular Medicine 5 (2018): 159. 30456215 10.3389/fcvm.2018.00159 PMC6230589

[33] C. Pei , X. Gong , and Y. Zhang , “LncRNA MALAT‐1 Promotes Growth and Metastasis of Epithelial Ovarian Cancer via Sponging Microrna‐22,” American Journal of Translational Research 12, no. 11 (2020): 6977–6987. 33312345 PMC7724350

[34] Z. Chen , D. Tian , X. Chen , et al., “Super‐Enhancer‐Driven lncRNA LIMD1‐AS1 Activated by CDK7 Promotes Glioma Progression,” Cell Death & Disease 14, no. 6 (2023): 383. 37385987 10.1038/s41419-023-05892-z PMC10310775

[35] J. F. Xiang , Q. F. Yin , T. Chen , et al., “Human Colorectal Cancer‐Specific CCAT1‐L lncRNA Regulates Long‐Range Chromatin Interactions at the MYC Locus,” Cell Research 24, no. 5 (2014): 513–531. 24662484 10.1038/cr.2014.35 PMC4011346

[36] Z. Cai , C. Cao , L. Ji , et al., “RIC‐Seq for Global In Situ Profiling of RNA‐RNA Spatial Interactions,” Nature 582, no. 7812 (2020): 432–437. 32499643 10.1038/s41586-020-2249-1

[37] J. W. Blayney , H. Francis , A. Rampasekova , et al., “Super‐Enhancers Include Classical Enhancers and Facilitators to Fully Activate Gene Expression,” Cell 186, no. 26 (2023): 5826–5839.e18. 38101409 10.1016/j.cell.2023.11.030 PMC10858684

[38] J. Zhou , S. H. M. Toh , T. K. Tan , et al., “Super‐Enhancer‐Driven TOX2 Mediates Oncogenesis in Natural Killer/T Cell Lymphoma,” Molecular Cancer 22, no. 1 (2023): 69. 37032358 10.1186/s12943-023-01767-1 PMC10084643

[39] L. A. Boyer , T. I. Lee , M. F. Cole , et al., “Core Transcriptional Regulatory Circuitry in Human Embryonic Stem Cells,” Cell 122, no. 6 (2005): 947–956. 16153702 10.1016/j.cell.2005.08.020 PMC3006442

[40] X. Pan , X. Li , J. Sun , et al., “Enhancer Methylation Dynamics Drive Core Transcriptional Regulatory Circuitry in Pan‐Cancer,” Oncogene 41, no. 26 (2022): 3474–3484. 35655092 10.1038/s41388-022-02359-x

[41] Y. Feng , T. Zhang , Z. Zhang , et al., “The Super‐Enhancer‐Driven lncRNA LINC00880 Acts as a Scaffold Between CDK1 and PRDX1 to Sustain the Malignance of Lung Adenocarcinoma,” Cell Death & Disease 14, no. 8 (2023): 551. 37620336 10.1038/s41419-023-06047-w PMC10449921

[42] T. Zhang , X. Song , Z. Zhang , et al., “Aberrant Super‐Enhancer Landscape Reveals Core Transcriptional Regulatory Circuitry in Lung Adenocarcinoma,” Oncogene 9, no. 10 (2020): 92. 10.1038/s41389-020-00277-9 PMC7568720 33070167

[43] Q. Shen , R. Wang , X. Liu , et al., “HSF1 Stimulates Glutamine Transport by Super‐Enhancer‐Driven lncRNA LINC00857 in Colorectal Cancer,” Cancers (Basel) 14, no. 16 (2022): 3855. 10.3390/cancers14163855 PMC9406190 36010849

[44] X. Q. Yuan , N. Zhou , J. P. Wang , et al., “Anchoring Super‐Enhancer‐Driven Oncogenic lncRNAs for Anti‐Tumor Therapy in Hepatocellular Carcinoma,” Molecular Therapy 31, no. 6 (2023): 1756–1774. 36461633 10.1016/j.ymthe.2022.11.013 PMC10277835

[45] W. Liang , C. Shi , W. Hong , et al., “Super‐Enhancer‐Driven lncRNA‐DAW Promotes Liver Cancer Cell Proliferation Through Activation of Wnt/β‐Catenin Pathway,” Molecular Therapy‐Nucleic Acids 26 (2021): 1351–1363. 34853732 10.1016/j.omtn.2021.10.028 PMC8608597

[46] J. Li , J. Wang , Y. Wang , X. Zhao , and T. Su , “E2F1 Combined With LINC01004 Super‐Enhancer to Promote Hepatocellular Carcinoma Cell Proliferation and Metastasis,” Clinical Epigenetics 15, no. 1 (2023): 17. 36721155 10.1186/s13148-023-01428-6 PMC9887888

[47] T. Su , N. Zhang , T. Wang , et al., “Super Enhancer‐Regulated LncRNA LINC01089 Induces Alternative Splicing of DIAPH3 to Drive Hepatocellular Carcinoma Metastasis,” Cancer Research 83, no. 24 (2023): 4080–4094. 37756562 10.1158/0008-5472.CAN-23-0544

[48] E. Bian , X. Chen , L. Cheng , et al., “Super‐Enhancer‐Associated TMEM44‐AS1 Aggravated Glioma Progression by Forming a Positive Feedback Loop With Myc,” Journal of Experimental & Clinical Cancer Research 40, no. 1 (2021): 337. 34696771 10.1186/s13046-021-02129-9 PMC8543865

[49] S. Dong , W. Wang , Z. Liao , Y. Fan , Q. Wang , and L. Zhang , “MYC‐Activated LINC00607 Promotes Hepatocellular Carcinoma Progression by Regulating the miR‐584‐3p/ROCK1 Axis,” Journal of Gene Medicine 25, no. 4 (2023): e3477. 36740760 10.1002/jgm.3477

[50] X. Zhang , Q. Zhang , and G. Liu , “Genome‐Wide Analysis of the FOXA1 Transcriptional Regulatory Network Identifies Super Enhancer Associated LncRNAs in Tamoxifen Resistance,” Frontiers in Genetics 13 (2022): 992444. 36204307 10.3389/fgene.2022.992444 PMC9530462

[51] Y. Zhang , Y. X. Huang , D. L. Wang , et al., “LncRNA DSCAM‐AS1 Interacts With YBX1 to Promote Cancer Progression by Forming a Positive Feedback Loop That Activates FOXA1 Transcription Network,” Theranostics 10, no. 23 (2020): 10823–10837. 32929382 10.7150/thno.47830 PMC7482804

[52] M. Pariollaud , L. H. Ibrahim , E. Irizarry , et al., “Circadian Disruption Enhances HSF1 Signaling and Tumorigenesis in Kras‐Driven Lung Cancer,” Science Advances 8, no. 39 (2022): eabo1123. 36170373 10.1126/sciadv.abo1123 PMC9519049

[53] Y. Chin , K. E. Gumilar , X. G. Li , et al., “Targeting HSF1 for Cancer Treatment: Mechanisms and Inhibitor Development,” Theranostics 13, no. 7 (2023): 2281–2300. 37153737 10.7150/thno.82431 PMC10157728

[54] M. Huang , W. Dong , R. Xie , et al., “HSF1 Facilitates the Multistep Process of Lymphatic Metastasis in Bladder Cancer via a Novel PRMT5‐WDR5‐Dependent Transcriptional Program,” Cancer Communications 42, no. 5 (2022): 447–470. 35434944 10.1002/cac2.12284 PMC9118058

[55] C. Dai and S. B. Sampson , “HSF1: Guardian of Proteostasis in Cancer,” Trends in Cell Biology 26, no. 1 (2016): 17–28. 26597576 10.1016/j.tcb.2015.10.011 PMC4722819

[56] C. Dai , L. Whitesell , A. B. Rogers , and S. Lindquist , “Heat Shock Factor 1 Is a Powerful Multifaceted Modifier of Carcinogenesis,” Cell 130, no. 6 (2007): 1005–1018. 17889646 10.1016/j.cell.2007.07.020 PMC2586609

[57] O. Meurette and P. Mehlen , “Notch Signaling in the Tumor Microenvironment,” Cancer Cell 34, no. 4 (2018): 536–548. 30146333 10.1016/j.ccell.2018.07.009

[58] L. Chen , R. P. Vasoya , N. H. Toke , et al., “HNF4 Regulates Fatty Acid Oxidation and Is Required for Renewal of Intestinal Stem Cells in Mice,” Gastroenterology 158, no. 4 (2020): 985–999.e9. 31759926 10.1053/j.gastro.2019.11.031 PMC7062567

[59] D. A. Michelson , C. Zuo , M. Verzi , C. Benoist , and D. Mathis , “Hnf4 Activates Mimetic‐Cell Enhancers to Recapitulate Gut and Liver Development Within the Thymus,” Journal of Experimental Medicine 220, no. 10 (2023): e20230461. 37399024 10.1084/jem.20230461 PMC10318407

[60] X. Zheng , M. Huang , L. Xing , et al., “The circRNA circSEPT9 Mediated by E2F1 and EIF4A3 Facilitates the Carcinogenesis and Development of Triple‐Negative Breast Cancer,” Molecular Cancer 19, no. 1 (2020): 73. 32264877 10.1186/s12943-020-01183-9 PMC7137343

[61] U. Wellner , J. Schubert , U. C. Burk , et al., “The EMT‐Activator ZEB1 Promotes Tumorigenicity by Repressing Stemness‐Inhibiting microRNAs,” Nature Cell Biology 11, no. 12 (2009): 1487–1495. 19935649 10.1038/ncb1998

[62] N. Ibrahim , L. He , C. O. Leong , et al., “BRCA1‐Associated Epigenetic Regulation of p73 Mediates an Effector Pathway for Chemosensitivity in Ovarian Carcinoma,” Cancer Research 70, no. 18 (2010): 7155–7165. 20807817 10.1158/0008-5472.CAN-10-0668 PMC2940979

[63] Y. Haddad , W. Choi , and D. J. McConkey , “Delta‐Crystallin Enhancer Binding Factor 1 Controls the Epithelial to Mesenchymal Transition Phenotype and Resistance to the Epidermal Growth Factor Receptor Inhibitor Erlotinib in Human Head and Neck Squamous Cell Carcinoma Lines,” Clinical Cancer Research 15, no. 2 (2009): 532–542. 19147758 10.1158/1078-0432.CCR-08-1733 PMC2729136

[64] C. Polytarchou , D. Iliopoulos , and K. Struhl , “An Integrated Transcriptional Regulatory Circuit That Reinforces the Breast Cancer Stem Cell State,” Proceedings of the National Academy of Sciences of the United States of America 109, no. 36 (2012): 14470–14475. 22908280 10.1073/pnas.1212811109 PMC3437881

[65] A. J. Bass , H. Watanabe , C. H. Mermel , et al., “SOX2 Is an Amplified Lineage‐Survival Oncogene in Lung and Esophageal Squamous Cell Carcinomas,” Nature Genetics 41, no. 11 (2009): 1238–1242. 19801978 10.1038/ng.465 PMC2783775

[66] L. Ha , R. M. Ponnamperuma , S. Jay , M. S. Ricci , and W. C. Weinberg , “Dysregulated ΔNp63α Inhibits Expression of Ink4a/Arf, Blocks Senescence, and Promotes Malignant Conversion of Keratinocytes,” PLoS One 6, no. 7 (2011): e21877. 21789189 10.1371/journal.pone.0021877 PMC3137598

[67] W. M. Keyes , M. Pecoraro , V. Aranda , et al., “ΔNp63α Is an Oncogene That Targets Chromatin Remodeler Lsh to Drive Skin Stem Cell Proliferation and Tumorigenesis,” Cell Stem Cell 8, no. 2 (2011): 164–176. 21295273 10.1016/j.stem.2010.12.009 PMC4373450

[68] K. Balmanno and S. J. Cook , “Tumour Cell Survival Signalling by the ERK1/2 Pathway,” Cell Death and Differentiation 16, no. 3 (2009): 368–377. 18846109 10.1038/cdd.2008.148

[69] L. C. Cantley , “The Phosphoinositide 3‐Kinase Pathway,” Science 296, no. 5573 (2002): 1655–1657. 12040186 10.1126/science.296.5573.1655

[70] H. Chen , H. Liu , and G. Qing , “Targeting Oncogenic Myc as a Strategy for Cancer Treatment,” Signal Transduction and Targeted Therapy 3 (2018): 5. 29527331 10.1038/s41392-018-0008-7 PMC5837124

[71] A. Baluapuri , E. Wolf , and M. Eilers , “Target Gene‐Independent Functions of MYC Oncoproteins,” Nature Reviews Molecular Cell Biology 21, no. 5 (2020): 255–267. 32071436 10.1038/s41580-020-0215-2 PMC7611238

[72] S. Yang , Y. Liu , M. Y. Li , et al., “FOXP3 Promotes Tumor Growth and Metastasis by Activating Wnt/β‐Catenin Signaling Pathway and EMT in Non‐Small Cell Lung Cancer,” Molecular Cancer 16, no. 1 (2017): 124. 28716029 10.1186/s12943-017-0700-1 PMC5514503

[73] J. Peng , S. Yang , C. S. H. Ng , and G. G. Chen , “The Role of FOXP3 in Non‐Small Cell Lung Cancer and Its Therapeutic Potentials,” Pharmacology & Therapeutics 241 (2023): 108333. 36528259 10.1016/j.pharmthera.2022.108333

[74] H. Jia , H. Qi , Z. Gong , et al., “The Expression of FOXP3 and Its Role in Human Cancers,” Biochimica et Biophysica Acta. Reviews on Cancer 1871, no. 1 (2019): 170–178. 30630091 10.1016/j.bbcan.2018.12.004

[75] A. Merlo , P. Casalini , M. L. Carcangiu , et al., “FOXP3 Expression and Overall Survival in Breast Cancer,” Journal of Clinical Oncology 27, no. 11 (2009): 1746–1752. 19255331 10.1200/JCO.2008.17.9036

[76] X. Cui , C. Zhang , Z. Xu , et al., “Dual CRISPR Interference and Activation for Targeted Reactivation of X‐Linked Endogenous FOXP3 in Human Breast Cancer Cells,” Molecular Cancer 21, no. 1 (2022): 38. 35130925 10.1186/s12943-021-01472-x PMC8819949

[77] E. J. Adams , W. R. Karthaus , E. Hoover , et al., “FOXA1 Mutations Alter Pioneering Activity, Differentiation and Prostate Cancer Phenotypes,” Nature 571, no. 7765 (2019): 408–412. 31243370 10.1038/s41586-019-1318-9 PMC6661172

[78] M. Teng , S. Zhou , C. Cai , M. Lupien , and H. H. He , “Pioneer of Prostate Cancer: Past, Present and the Future of FOXA1,” Protein & Cell 12, no. 1 (2021): 29–38. 32946061 10.1007/s13238-020-00786-8 PMC7815845

[79] J. I. Warrick , W. Hu , H. Yamashita , et al., “FOXA1 Repression Drives Lineage Plasticity and Immune Heterogeneity in Bladder Cancers With Squamous Differentiation,” Nature Communications 13, no. 1 (2022): 6575. 10.1038/s41467-022-34251-3 PMC9630410 36323682

[80] Y. S. Niknafs , S. Han , T. Ma , et al., “The lncRNA Landscape of Breast Cancer Reveals a Role for DSCAM‐AS1 in Breast Cancer Progression,” Nature Communications 7 (2016): 12791. 10.1038/ncomms12791 PMC5052669 27666543

[81] J. Liao and N. Xie , “Long Noncoding RNA DSCAM‐AS1 Functions AS an Oncogene in Non‐Small Cell Lung Cancer by Targeting BCL11A,” European Review for Medical and Pharmacological Sciences 23, no. 3 (2019): 1087–1092. 30779076 10.26355/eurrev_201902_16998

[82] X. Chen , C. C. Yan , X. Zhang , and Z. H. You , “Long Non‐Coding RNAs and Complex Diseases: From Experimental Results to Computational Models,” Briefings in Bioinformatics 18, no. 4 (2017): 558–576. 27345524 10.1093/bib/bbw060 PMC5862301

[83] Y. T. Tan , J. F. Lin , T. Li , J. J. Li , R. H. Xu , and H. Q. Ju , “LncRNA‐Mediated Posttranslational Modifications and Reprogramming of Energy Metabolism in Cancer,” Cancer Communications 41, no. 2 (2021): 109–120. 33119215 10.1002/cac2.12108 PMC7896749

[84] Q. Jia , Y. Tan , Y. Li , Y. Wu , J. Wang , and F. Tang , “JUN‐Induced Super‐Enhancer RNA Forms R‐Loop to Promote Nasopharyngeal Carcinoma Metastasis,” Cell Death & Disease 14, no. 7 (2023): 459. 37479693 10.1038/s41419-023-05985-9 PMC10361959

[85] F. Yao , Q. Wang , and Q. Wu , “The Prognostic Value and Mechanisms of lncRNA UCA1 in Human Cancer,” Cancer Management and Research 11 (2019): 7685–7696. 31616184 10.2147/CMAR.S200436 PMC6698587

[86] Z. Bian , L. Jin , J. Zhang , et al., “LncRNA‐UCA1 Enhances Cell Proliferation and 5‐Fluorouracil Resistance in Colorectal Cancer by Inhibiting miR‐204‐5p,” Scientific Reports 6 (2016): 23892. 27046651 10.1038/srep23892 PMC4820696

[87] X. Lin , T. J. Spindler , M. A. de Souza Fonseca , et al., “Super‐Enhancer‐Associated LncRNA UCA1 Interacts Directly With AMOT to Activate YAP Target Genes in Epithelial Ovarian Cancer,” iScience 17 (2019): 242–255. 31307004 10.1016/j.isci.2019.06.025 PMC6629722

[88] L. Yan , H. Chen , L. Tang , P. Jiang , and F. Yan , “Super‐Enhancer‐Associated Long Noncoding RNA AC005592.2 Promotes Tumor Progression by Regulating OLFM4 in Colorectal Cancer,” BMC Cancer 21, no. 1 (2021): 187. 33622275 10.1186/s12885-021-07900-x PMC7903608

[89] Z. Yang , Y. Zheng , H. Wu , et al., “Integrative Analysis of a Novel Super‐Enhancer‐Associated lncRNA Prognostic Signature and Identifying LINC00945 in Aggravating Glioma Progression,” Human Genomics 17, no. 1 (2023): 33. 37004060 10.1186/s40246-023-00480-w PMC10064652

[90] V. M. Conn , M. Gabryelska , J. Toubia , et al., “Circular RNAs Drive Oncogenic Chromosomal Translocations Within the MLL Recombinome in Leukemia,” Cancer Cell 41, no. 7 (2023): 1309–1326.e10. 37295428 10.1016/j.ccell.2023.05.002

[91] F. L. Meng , Z. du , A. Federation , et al., “Convergent Transcription at Intragenic Super‐Enhancers Targets AID‐Initiated Genomic Instability,” Cell 159, no. 7 (2014): 1538–1548. 25483776 10.1016/j.cell.2014.11.014 PMC4322776

[92] G. Rothschild and U. Basu , “Lingering Questions About Enhancer RNA and Enhancer Transcription‐Coupled Genomic Instability,” Trends in Genetics 33, no. 2 (2017): 143–154. 28087167 10.1016/j.tig.2016.12.002 PMC5291171

[93] Z. Lu , N. R. Pannunzio , H. A. Greisman , D. Casero , C. Parekh , and M. R. Lieber , “Convergent BCL6 and lncRNA Promoters Demarcate the Major Breakpoint Region for BCL6 Translocations,” Blood 126, no. 14 (2015): 1730–1731. 26276666 10.1182/blood-2015-07-657999 PMC4591794

[94] M. R. Lieber , “Mechanisms of Human Lymphoid Chromosomal Translocations,” Nature Reviews Cancer 16, no. 6 (2016): 387–398. 27220482 10.1038/nrc.2016.40 PMC5336345

[95] N. R. Pannunzio and M. R. Lieber , “RNA Polymerase Collision Versus DNA Structural Distortion: Twists and Turns Can Cause Break Failure,” Molecular Cell 62, no. 3 (2016): 327–334. 27153532 10.1016/j.molcel.2016.03.034 PMC4860253

[96] D. Zhou , S. He , D. Zhang , et al., “LINC00857 Promotes Colorectal Cancer Progression by Sponging miR‐150‐5p and Upregulating HMGB3 (High Mobility Group Box 3) Expression,” Bioengineered 12, no. 2 (2021): 12107–12122. 34753396 10.1080/21655979.2021.2003941 PMC8810051

[97] Q. Shen , H. Zhou , M. Zhang , et al., “Super Enhancer‐LncRNA SENCR Promoted Cisplatin Resistance and Growth of NSCLC Through Upregulating FLI1,” Journal of Clinical Laboratory Analysis 36, no. 6 (2022): e24460. 35500152 10.1002/jcla.24460 PMC9169188

[98] C. T. Ong and V. G. Corces , “CTCF: An Architectural Protein Bridging Genome Topology and Function,” Nature Reviews Genetics 15, no. 4 (2014): 234–246. 10.1038/nrg3663 PMC4610363 24614316

[99] J. P. Wells , J. White , and P. C. Stirling , “R Loops and Their Composite Cancer Connections,” Trends Cancer 5, no. 10 (2019): 619–631. 31706509 10.1016/j.trecan.2019.08.006

[100] A. Aguilera and T. García‐Muse , “R Loops: From Transcription Byproducts to Threats to Genome Stability,” Molecular Cell 46, no. 2 (2012): 115–124. 22541554 10.1016/j.molcel.2012.04.009

[101] T. A. Cooper , L. Wan , and G. Dreyfuss , “RNA and Disease,” Cell 136, no. 4 (2009): 777–793. 19239895 10.1016/j.cell.2009.02.011 PMC2866189

[102] A. G. Matera and Z. Wang , “A Day in the Life of the Spliceosome,” Nature Reviews Molecular Cell Biology 15, no. 2 (2014): 108–121. 24452469 10.1038/nrm3742 PMC4060434

[103] C. P. Bracken , H. S. Scott , and G. J. Goodall , “A Network‐Biology Perspective of microRNA Function and Dysfunction in Cancer,” Nature Reviews Genetics 17, no. 12 (2016): 719–732. 10.1038/nrg.2016.134 27795564

[104] V. O. Wickramasinghe and R. A. Laskey , “Control of Mammalian Gene Expression by Selective mRNA Export,” Nature Reviews Molecular Cell Biology 16, no. 7 (2015): 431–442. 26081607 10.1038/nrm4010

[105] Y. Shi , “Mechanistic Insights Into Precursor Messenger RNA Splicing by the Spliceosome,” Nature Reviews Molecular Cell Biology 18, no. 11 (2017): 655–670. 28951565 10.1038/nrm.2017.86

[106] G. J. Goodall and V. O. Wickramasinghe , “RNA in Cancer,” Nature Reviews Cancer 21, no. 1 (2021): 22–36. 33082563 10.1038/s41568-020-00306-0

[107] E. Wang , S. X. Lu , A. Pastore , et al., “Targeting an RNA‐Binding Protein Network in Acute Myeloid Leukemia,” Cancer Cell 35, no. 3 (2019): 369–384.e7. 30799057 10.1016/j.ccell.2019.01.010 PMC6424627

[108] J. N. Vo , M. Cieslik , Y. Zhang , et al., “The Landscape of Circular RNA in Cancer,” Cell 176, no. 4 (2019): 869–881.e13. 30735636 10.1016/j.cell.2018.12.021 PMC6601354

[109] H. Dvinge , E. Kim , O. Abdel‐Wahab , and R. K. Bradley , “RNA Splicing Factors as Oncoproteins and Tumour Suppressors,” Nature Reviews Cancer 16, no. 7 (2016): 413–430. 27282250 10.1038/nrc.2016.51 PMC5094465

[110] K. Yoshida , M. Sanada , Y. Shiraishi , et al., “Frequent Pathway Mutations of Splicing Machinery in Myelodysplasia,” Nature 478, no. 7367 (2011): 64–69. 21909114 10.1038/nature10496

[111] Y. Tay , J. Rinn , and P. Pandolfi , “The Multilayered Complexity of ceRNA Crosstalk and Competition,” Nature 505, no. 7483 (2014): 344–352. 24429633 10.1038/nature12986 PMC4113481

[112] M. Cesana , D. Cacchiarelli , I. Legnini , et al., “A Long Noncoding RNA Controls Muscle Differentiation by Functioning as a Competing Endogenous RNA,” Cell 147, no. 2 (2011): 358–369. 22000014 10.1016/j.cell.2011.09.028 PMC3234495

[113] X. Z. Yang , T. T. Cheng , Q. J. He , et al., “LINC01133 as ceRNA Inhibits Gastric Cancer Progression by Sponging miR‐106a‐3p to Regulate APC Expression and the Wnt/β‐Catenin Pathway,” Molecular Cancer 17, no. 1 (2018): 126. 30134915 10.1186/s12943-018-0874-1 PMC6106894

[114] Z. T. Yao , Y. M. Yang , M. M. Sun , et al., “New Insights Into the Interplay Between Long Non‐Coding RNAs and RNA‐Binding Proteins in Cancer,” Cancer Communications 42, no. 2 (2022): 117–140. 35019235 10.1002/cac2.12254 PMC8822594

[115] J. M. Engreitz , N. Ollikainen , and M. Guttman , “Long Non‐Coding RNAs: Spatial Amplifiers That Control Nuclear Structure and Gene Expression,” Nature Reviews Molecular Cell Biology 17, no. 12 (2016): 756–770. 27780979 10.1038/nrm.2016.126

[116] X. Zhang , M. Liu , Z. Li , L. Zhuo , X. Fu , and Q. Zou , “Fusion of Multi‐Source Relationships and Topology to Infer lncRNA‐Protein Interactions,” Molecular Therapy ‐ Nucleic Acids 35, no. 2 (2024): 102187. 38706631 10.1016/j.omtn.2024.102187 PMC11066462

[117] Z. Zhou , Z. du , J. Wei , et al., “MHAM‐NPI: Predicting ncRNA‐Protein Interactions Based on Multi‐Head Attention Mechanism,” Computers in Biology and Medicine 163 (2023): 107143. 37339574 10.1016/j.compbiomed.2023.107143

[118] J. Wei , L. Zhuo , S. Pan , X. Lian , X. Yao , and X. Fu , “HeadTailTransfer: An Efficient Sampling Method to Improve the Performance of Graph Neural Network Method in Predicting Sparse ncRNA‐Protein Interactions,” Computers in Biology and Medicine 157 (2023): 106783. 36958237 10.1016/j.compbiomed.2023.106783

[119] W. Liu , T. Tang , X. Lu , X. Fu , Y. Yang , and L. Peng , “MPCLCDA: Predicting circRNA‐Disease Associations by Using Automatically Selected Meta‐Path and Contrastive Learning,” Briefings in Bioinformatics 24, no. 4 (2023): bbad227. 37328701 10.1093/bib/bbad227

[120] Z. Li , P. Hou , D. Fan , et al., “The Degradation of EZH2 Mediated by lncRNA ANCR Attenuated the Invasion and Metastasis of Breast Cancer,” Cell Death and Differentiation 24, no. 1 (2017): 59–71. 27716745 10.1038/cdd.2016.95 PMC5260507

[121] L. Fu , C. Zhang , L. Y. Zhang , et al., “Wnt2 Secreted by Tumour Fibroblasts Promotes Tumour Progression in Oesophageal Cancer by Activation of the Wnt/β‐Catenin Signalling Pathway,” Gut 60, no. 12 (2011): 1635–1643. 21672941 10.1136/gut.2011.241638

[122] M. Katoh , “Frequent Up‐Regulation of WNT2 in Primary Gastric Cancer and Colorectal Cancer,” International Journal of Oncology 19, no. 5 (2001): 1003–1007. 11605001 10.3892/ijo.19.5.1003

[123] D. T. Bravo , Y. L. Yang , K. Kuchenbecker , et al., “Frizzled‐8 Receptor Is Activated by the Wnt‐2 Ligand in Non‐Small Cell Lung Cancer,” BMC Cancer 13 (2013): 316. 23815780 10.1186/1471-2407-13-316 PMC3707790

[124] B. Z. Vider , A. Zimber , E. Chastre , et al., “Evidence for the Involvement of the Wnt 2 Gene in Human Colorectal Cancer,” Oncogene 12, no. 1 (1996): 153–158. 8552386

[125] S. Piccolo , S. Dupont , and M. Cordenonsi , “The Biology of YAP/TAZ: Hippo Signaling and Beyond,” Physiological Reviews 94, no. 4 (2014): 1287–1312. 25287865 10.1152/physrev.00005.2014

[126] O. Morana , W. Wood , and C. D. Gregory , “The Apoptosis Paradox in Cancer,” International Journal of Molecular Sciences 23, no. 3 (2022): 1328. 35163253 10.3390/ijms23031328 PMC8836235

[127] G. Kroemer and J. Pouyssegur , “Tumor Cell Metabolism: Cancer's Achilles' Heel,” Cancer Cell 13, no. 6 (2008): 472–482. 18538731 10.1016/j.ccr.2008.05.005

[128] S. S. Ousman and P. Kubes , “Immune Surveillance in the Central Nervous System,” Nature Neuroscience 15, no. 8 (2012): 1096–1101. 22837040 10.1038/nn.3161 PMC7097282

[129] A. Ribas and J. D. Wolchok , “Cancer Immunotherapy Using Checkpoint Blockade,” Science 359, no. 6382 (2018): 1350–1355. 29567705 10.1126/science.aar4060 PMC7391259

[130] Y. Xu , Y. Wu , S. Zhang , et al., “A Tumor‐Specific Super‐Enhancer Drives Immune Evasion by Guiding Synchronous Expression of PD‐L1 and PD‐L2,” Cell Reports 29, no. 11 (2019): 3435–3447.e4. 31825827 10.1016/j.celrep.2019.10.093

[131] H. Noriega‐Guerra and V. M. Freitas , “Extracellular Matrix Influencing HGF/c‐MET Signaling Pathway: Impact on Cancer Progression,” International Journal of Molecular Sciences 19, no. 11 (2018): 3300. 30352967 10.3390/ijms19113300 PMC6274944

[132] I. M. Moya and G. Halder , “Hippo‐YAP/TAZ Signalling in Organ Regeneration and Regenerative Medicine,” Nature Reviews Molecular Cell Biology 20, no. 4 (2019): 211–226. 30546055 10.1038/s41580-018-0086-y

[133] L. T. Y. Ho , N. Skiba , C. Ullmer , and P. V. Rao , “Lysophosphatidic Acid Induces ECM Production via Activation of the Mechanosensitive YAP/TAZ Transcriptional Pathway in Trabecular Meshwork Cells,” Investigative Ophthalmology & Visual Science 59, no. 5 (2018): 1969–1984. 29677358 10.1167/iovs.17-23702 PMC5896423

[134] C. Zhou , S. R. York , J. Y. Chen , et al., “Long Noncoding RNAs Expressed in Human Hepatic Stellate Cells Form Networks With Extracellular Matrix Proteins,” Genome Medicine 8, no. 1 (2016): 31. 27007663 10.1186/s13073-016-0285-0 PMC4804564

[135] Y. Tan , Y. Li , and F. Tang , “Oncogenic seRNA Functional Activation: A Novel Mechanism of Tumorigenesis,” Molecular Cancer 19, no. 1 (2020): 74. 32278350 10.1186/s12943-020-01195-5 PMC7149907

[136] C. Viallard and B. Larrivée , “Tumor Angiogenesis and Vascular Normalization: Alternative Therapeutic Targets,” Angiogenesis 20, no. 4 (2017): 409–426. 28660302 10.1007/s10456-017-9562-9

[137] H. Wei , F. Wang , Y. Wang , et al., “Verteporfin Suppresses Cell Survival, Angiogenesis and Vasculogenic Mimicry of Pancreatic Ductal Adenocarcinoma via Disrupting the YAP‐TEAD Complex,” Cancer Science 108, no. 3 (2017): 478–487. 28002618 10.1111/cas.13138 PMC5378285

[138] Y. Hu , L. Lei , L. Jiang , et al., “LncRNA UCA1 Promotes Keratinocyte‐Driven Inflammation via Suppressing METTL14 and Activating the HIF‐1α/NF‐κB Axis in Psoriasis,” Cell Death & Disease 14, no. 4 (2023): 279. 37076497 10.1038/s41419-023-05790-4 PMC10115875

[139] X. P. Li , J. Qu , X. Q. Teng , et al., “The Emerging Role of Super‐Enhancers as Therapeutic Targets in the Digestive System Tumors,” International Journal of Biological Sciences 19, no. 4 (2023): 1036–1048. 36923930 10.7150/ijbs.78535 PMC10008685

[140] Y. Wang , H. Nie , X. He , et al., “The Emerging Role of Super Enhancer‐Derived Noncoding RNAs in Human Cancer,” Theranostics 10, no. 24 (2020): 11049–11062. 33042269 10.7150/thno.49168 PMC7532672

[141] S. Xu , L. Wan , H. Yin , et al., “Long Noncoding RNA Linc00152 Functions as a Tumor Propellant in Pan‐Cancer,” Cellular Physiology and Biochemistry 44 (2017): 2476–2490. 29268251 10.1159/000486170

[142] H. Zhou , S. C. S. Schmidt , S. Jiang , et al., “Epstein‐Barr Virus Oncoprotein Super‐Enhancers Control B Cell Growth,” Cell Host & Microbe 17, no. 2 (2015): 205–216. 25639793 10.1016/j.chom.2014.12.013 PMC4539236

[143] P. Eliades , B. J. Abraham , Z. Ji , et al., “High MITF Expression Is Associated With Super‐Enhancers and Suppressed by CDK7 Inhibition in Melanoma,” Journal of Investigative Dermatology 138, no. 7 (2018): 1582–1590. 29408204 10.1016/j.jid.2017.09.056 PMC6019629

[144] H. E. Pelish , B. B. Liau , I. I. Nitulescu , et al., “Mediator Kinase Inhibition Further Activates Super‐Enhancer‐Associated Genes in AML,” Nature 526, no. 7572 (2015): 273–276. 26416749 10.1038/nature14904 PMC4641525

